# Engineering Allosteric Transcription Factor-Based Biosensors: Advances and Prospects for Modern Food Contaminant Monitoring

**DOI:** 10.3390/foods15030597

**Published:** 2026-02-06

**Authors:** Xinyue Lan, Ziying Zhou, Yanger Liu, Xiangyang Li, Wenbiao Shi, Longjiao Zhu, Wentao Xu

**Affiliations:** 1 Food Laboratory of Zhongyuan, Key Laboratory of Precision Nutrition and Food Quality, Beijing Laboratory for Food Quality and Safety, Key Laboratory of Safety Assessment of Genetically, Modified Organism (Food Safety), Department of Nutrition and Health, China Agricultural University, Beijing 100193, China; 2 Beijing Laboratory of Food Quality and Safety, Beijing Key Laboratory of Agricultural Product Detection and Control of Spoilage Organisms and Pesticide Residue, Key Laboratory of Agricultural Product Processing and Quality Control (Co-construction by Ministry and Province), Faculty of Food Science and Engineering, Beijing University of Agriculture, Beijing 102206, China; 3 State Key Laboratory for Quality and Safety of Agro-Products, Institute of Quality Standards and Testing Technology for Agro-Products, Chinese Academy of Agricultural Sciences, Haidian, Beijing 100081, China

**Keywords:** allosteric transcription factor, in vitro biosensors, construction strategy, food safety detection, recognition element

## Abstract

Allosteric transcription factor (aTF)-based in vitro biosensors constitute a class of detection tools formed by the functional coupling of the ligand-binding domain of aTFs with a reporter system. Owing to advantages such as high specificity and sensitivity, these biosensors have emerged as a research hotspot in the field of modern food contaminant monitoring. Our work centers on the core aspect of engineering design and systematically elaborates on the modular design strategies for aTF-based in vitro biosensors, with a focus on the design principles of the molecular recognition system, signal amplification strategies, signal output systems, and sensing systems. Furthermore, the article summarizes the advances in the application of aTF biosensors for detecting various typical food contaminants and analyzes their performance advantages. Finally, in light of existing technical limitations, it prospectively discusses future directions for enhancing specificity, improving stability, and promoting commercial applications, aiming to provide a theoretical reference and application guidance for transitioning this technology from laboratory platforms to on-site real-time monitoring.

## 1. Introduction

**Figure 1:**
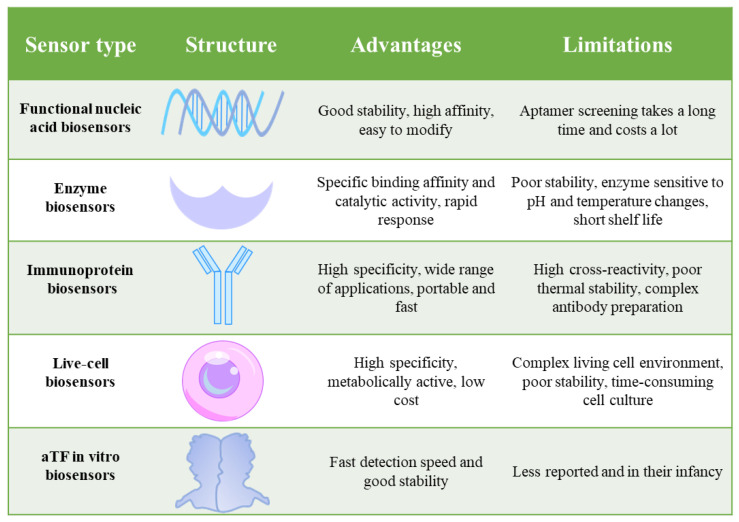
Comparison of the performance of different biosensors.

Food safety is an important and serious topic in modern society, which is related to everyone’s health and quality of life. With the acceleration of globalization and the rapid development of the food industry, food safety issues have become increasingly prominent, and frequent food safety incidents not only pose a threat to public health but also have far-reaching impacts on economic development and social stability. For example, residual effects of tetracycline can lead to impaired fetal development, gastrointestinal disturbances, and pro-inflammatory, cytotoxic and immunopathological effects on human health [1]. Therefore, it is necessary for us to carry out rapid and accurate detection of food safety. Currently, biosensors play a key role in the food industry. They mainly consist of a biomolecular recognition element, a signal converter and a signal output device [2]. Typically, biologically active substances, such as nucleic acids, enzymes, antibodies, antigens, cells, etc., are used as common recognition elements, which bind specifically to the target analyte or induce binding by affinity. Subsequently, a signal converter converts the biorecognition into measurable signals, such as optical, electrical, thermal, etc., and outputs them via a signal output device for detection purposes [3]. Various biosensors based on different components are being actively developed for fast and simple detection of contaminants in food. However, existing biosensors still have some limitations. For example, the process of generating and characterizing new antibodies in immuno-biosensors is time-consuming and complex [4]. In addition, enzyme biosensors face problems such as enzyme sensitivity to temperature and pH, as well as short shelf lives [5]. In recent years, researchers have found that the molecular recognition elements of biosensors are not limited to the several conventional substances mentioned above and that altered transcription factors (aTFs) can also be used as independent recognition elements. Biosensors based on aTFs have shown significant contributions in the fields of food safety, environmental monitoring and clinical diagnosis [6,7,8] (Figure 1). This development marks an emerging direction in the field of detection and opens a whole new avenue of biosensor development.

aTFs are widespread in nature and vary in their type and recognition of specific targets. Studies have shown that such proteins can be found in mammals, plants and microorganisms. On this basis, researchers further found that in prokaryotes, aTFs are regulatory proteins produced during bacterial evolution that can sense various stimuli from the external environment [9], and they are valued for their highly specific recognition of substrates. An aTF consists of a ligand-binding domain (LBD) and a DNA-binding domain (DBD). Binding of specific small molecules to the LBD induces conformational changes in the aTF that modulate the affinity of the DBD for the transcription factor binding site (TFBS), thereby altering the expression of downstream genes [10]. DBDs show significant similarity and conservation between different aTF families, whereas LBDs have greater variability and diversity, enabling aTFs to recognize and detect a wide range of potential effector molecules [11,12] Drawing upon the programmable nature of aTFs, their engineering applications are firmly grounded. Leveraging synthetic biology tools such as directed evolution, researchers can now tailor or optimize aTFs to recognize specific targets or enhance their performance. For instance, Cayron et al. [13] engineered a mutant of RcnR with high specificity for nickel ions, significantly lowering the detection limit to 80 nM. This demonstrates the feasibility of on-demand design of biosensing elements.

However, the ultimate performance of aTFs is highly dependent on the sensing platform in which they are deployed. Early research predominantly integrated aTFs into whole-cell biosensors (WCBs) [14]. While WCBs benefit from the full context of cellular reactions, their utility is severely constrained by the inherent complexities of living systems: demanding culture conditions, limited long-term stability, safety concerns related to genetically modified organisms (GMOs), and an inability to detect cytotoxic or membrane-impermeable analytes such as certain organophosphorus compounds [15,16].

Consequently, decoupling aTFs from cellular environments to construct in vitro detection systems has emerged as a critical strategy to overcome these limitations. Studies have confirmed that aTFs retain their core functions, namely, ligand recognition and conformational switching, even in well-defined buffered systems [17]. This property has enabled the development of aTF-based in vitro biosensors. By directly coupling purified aTF proteins with signaling reporter modules in a controlled in vitro environment, this system achieves highly sensitive and specific detection of targets while circumventing the inherent drawbacks of whole-cell platforms. This represents a significant step forward in transforming aTF technology into more stable, safer, and more versatile detection tools.

Up to now, several research teams at home and abroad have studied aTF biosensors in different dimensions. For example, Li’s team [17] summarized the research progress of aTF biosensors based on in vitro configurations and demonstrated their potential in in vitro applications. Jeon et al. [18] introduced the recent progress of transcription factor (TF)-based biosensors in pathogen detection. Meanwhile, Zhang et al., in ref. [19], reviewed the application of cell-free biosensors in environmental monitoring and health diagnosis. However, a dedicated and systematic review that details the construction strategies of aTF biosensors, with a specific focus on their design and application for food safety detection, remains lacking. Therefore, this review will aim to elaborate the core components of aTF in vitro biosensors, including the molecular recognition system, signal amplification strategy, signal output system, and sensing system. In addition, we will summarize the applications of aTF in vitro biosensors in food safety fields such as heavy metal ions, pesticide and veterinary drug residues, food additives, and foodborne pathogens, and discuss the future trends in this focused area. This work is expected to improve the theoretical foundation of aTF in vitro biosensors and promote their development and application.

### Literature Search Methodology

This work is based on a systematic search of the literature from databases including PubMed, Web of Science, Google Scholar, ScienceDirect, ACS Publications, and SpringerLink, covering publications from 2000 to 2025, with a particular emphasis on and preference for studies from the last decade (2015–2025) to capture the most recent advances. The search strategy focused on key terms such as ‘allosteric transcription factor (aTF) biosensors,’ ‘aTF-based in vitro biosensors,’ ’food contaminant detection biosensors,’ ‘heavy metal detection aTF,’ and ‘antibiotic biosensor transcription factor,’ among others related to food safety detection and biosensor engineering. Included studies were those that addressed the design, mechanism, or application of aTF-based in vitro biosensors for detecting food-related contaminants, providing a focused overview of current research trends and evidence.

## 2. Strategies for the Construction of aTF-Based In Vitro Biosensors

**Figure 2:**
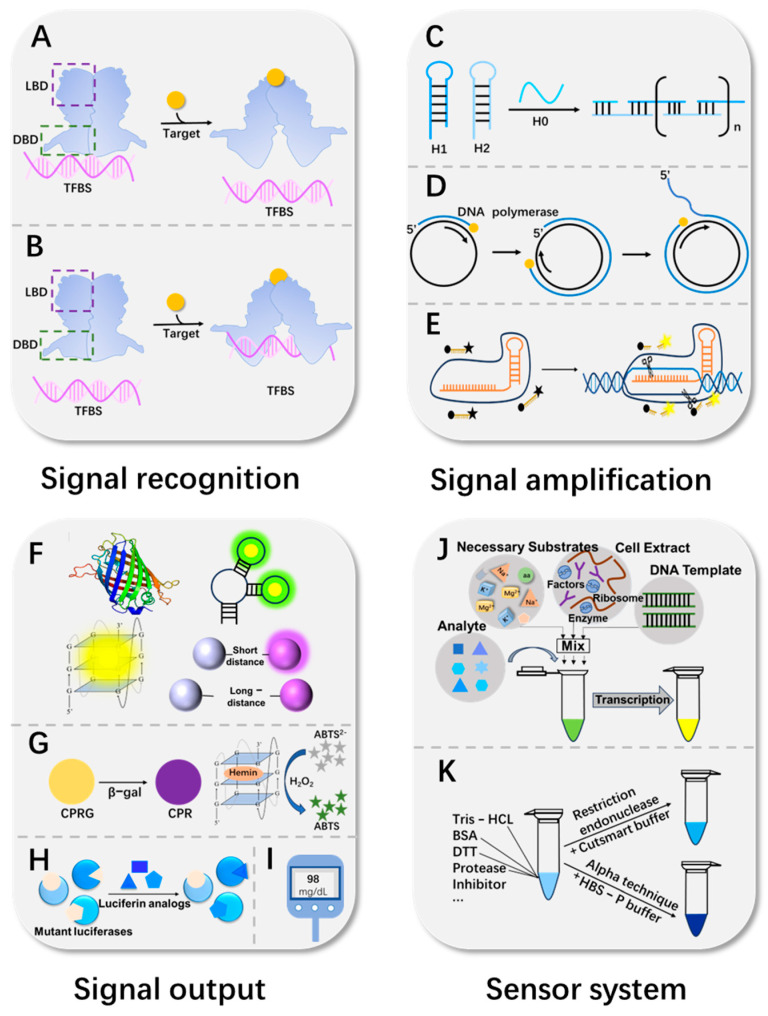
Schematic diagram of aTF biosensor mechanism. aTF biosensor working process includes signal recognition, signal amplification, signal output and specific sensing system. According to the effect of aTFs on gene regulation, these mechanisms can be classified into (**A**) inhibitory aTF regulatory mechanisms and (**B**) activating aTF regulatory mechanisms. An aTF can be combined with various signal amplification strategies such as (**C**) HCR technology, (**D**) RCA technology, and (**E**) CRISPR/Cas technology. Signal output systems convert detected signals into quantifiable results, mainly in the form of (**F**) fluorescence, (**G**) colorimetric, (**H**) bioluminescence and (**I**) electrical signals. In aTF in vitro biosensors, the sensing systems are mainly of two types: (**J**) cell-free systems and (**K**) compatible buffer systems.

The construction of aTF-based in vitro biosensors reflects the deep convergence between the fields of biology and engineering, which requires careful design and optimization of molecular recognition elements, signal amplification strategies and signal output systems. This mainly relies on an aTF-TFBS as the core biorecognition system, which significantly enhances the strength of the output detection signal through clever signal amplification strategies. During the reaction process, the sensor is usually placed in a specific sensing system, such as a compatible buffer system or a cell-free system, to ensure the stability and accuracy of the reaction. These elements work together to achieve high sensitivity, high specificity and rapid detection capabilities (Figure 2).

### 2.1. Molecular Recognition System

Molecular recognition of aTF biosensors is a complex and precise process centered on the interaction between the aTF, its specific TFBS, and the target molecule. The TFBS is usually a conserved short DNA sequence [20], serving as the target site recognized by the DBD of the aTF. The binding between an aTF and a TFBS is analogous to a lock and key, exhibiting a high degree of specificity. This ensures that biosensors can accurately identify and respond to target molecules in complex biological environments. When analytes are present, the LBD of an aTF is able to trigger a conformational change in the aTF itself upon recognition of a specific ligand, altering the affinity between the DBD and the TFBS, thus enabling precise control of gene expression. Not only do aTFs bind the binding motifs of double-stranded DNA (dsDNA), but they have also been found to recognize and capture SELEX-screened RNA as well [21]. Based on the effects on gene regulation, aTFs can be divided into two types: repressive aTFs and activating aTFs. In the field of food safety, most aTFs behave as the repressive type, such as MerR, TetR, MarR, etc. Taking the MerR family as an example, in the absence of a target molecule, the MerR family proteins act as inhibitors bound to the TFBS; however, when the target is recognized and bound, the binding of the DBD to the TFBS undergoes dissociation, thereby activating the output of the reporter element [22] (Figure 2A). In addition to this, there are a few activated aTFs, such as LasR in response to *Pseudomonas aeruginosa*. Binding of homoserine lactone (HSL) autoinducers stabilizes LasR, a member of the LuxR family, and promotes dimerization, and the resulting LasR N-(3-oxododecanoyl)-homoserine lactone autoinducer complex binds target DNA to activate gene transcription [23] (Figure 2B).

**Figure 3:**
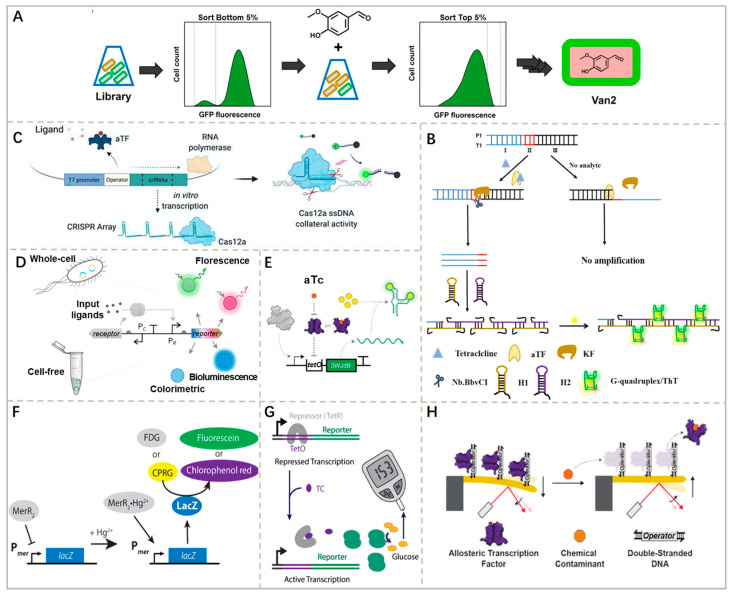
(**A**) Mutants with altered ligand specificity were enriched by fluorescence-activated cell sorting counter-selection using *E. coli* cultures with the PcaV DE library. Adapted from [8], with permission from the *Journal of Biological Engineering*, 2019. (**B**) Detection of TC using TetR as a recognition element controlling SDA and HCR. Adapted from [25], with permission from Elsevier, 2023. (**C**) Coupling of TetR-regulated CRISPR array-based in vitro transcriptional expression with Cas12a activity for TC detection. Adapted from [26], with permission from *Analytical Chemistry*, 2022. (**D**) Comprehensive analysis of fluorescent, colorimetric and bioluminescent gene reporters in whole-cell and cell-free expressed biosensor systems. Adapted from [27], with permission from the American Chemical Society, 2019. (**E**) Use of 3WJdB RNA aptamer as a signal output element in combination with DFHBI-1T dye to generate fluorescent signals for the detection of a wide range of water contaminants. Adapted from [6], with permission from *Nature Biotechnology*, 2020. (**F**) Monitoring of LacZ enzyme activity using chromogenic (CPRG) or fluorescent (FDC) substrates. Adapted from [28], with permission from the American Chemical Society, 2017. (**G**) Schematic of the alternative gene circuit for small-molecule detection based on the TetO/TetR induction system. Adapted from [29], with permission from *Nature Communications*, 2021. (**H**) Schematic of ultrasensitive water contaminant detection with aTF-interfaced microcantilevers. Adapted from [30], with permission from bioRxiv *Synthetic Biology*, 2024.

In current scientific research, most studied aTFs are naturally occurring and unmodified. However, the efficacy and regulatory ability of these natural aTFs are often limited in practical applications and cannot fully meet the actual detection needs. To solve these limitations, researchers within synthetic biology have begun employing a series of advanced biotechnological techniques for the artificial modification of aTFs. Through genetic engineering methods such as point mutation, gene knockout, gene insertion, etc., the coding genes of aTFs can be precisely modified to alter their recognition specificity, binding capacity or regulatory efficiency. In addition, the amino acid sequence of aTFs can be modified using directed evolution, protein engineering and other technological means to alter the function of its structural domains and optimize its interaction with target molecules [8] (Figure 3A). Furthermore, researchers have also used computer simulations to accurately simulate and predict the interactions between aTFs and target molecules to guide the direction of aTF modification [24].

Based on the above, in aTF-based molecular recognition systems, the synergistic strategy combining directed evolution of natural aTFs with site-specific immobilization emerges as the one most effective for signal monitoring. Directed evolution techniques, such as error-prone PCR, can remodel the ligand-binding domains of aTFs. When integrated with rational design guided by structural biology simulations, this approach significantly enhances both affinity and specificity. Furthermore, employing site-specific immobilization methods, such as the SpyTag/SpyCatcher system, prevents the blocking of active sites, thereby increasing resistance to matrix interference and extending storage stability.

### 2.2. Signal Amplification Strategies

Since biosensors are typically required to detect target molecules at very low concentrations, effective signal amplification strategies are particularly crucial. Combining a small-molecule recognition factor aTF with multiple signal amplification strategies can significantly improve the analytical performance of biosensors in terms of sensitivity and specificity. Within the specific framework of aTF-based biosensors, the core function of the signal amplification module is to capture and amplify the primary signal generated by the molecular recognition event. This signal fundamentally manifests as a change in the binding state between the aTF and its TFBS. The most direct physical carriers of this change are specific DNA sequences, such as the operator DNA released from the TFBS or the RNA products novelly transcribed from aTF-regulated promoters. Consequently, most signal amplification strategies are designed to target these nucleic acid products, achieving exponential signal growth through enzymatic or non-enzymatic reactions, thereby transforming a weak specific recognition event into a strong, readily detectable signal.

#### 2.2.1. Isothermal Amplification Technique

Isothermal amplification techniques achieve signal amplification by efficiently replicating target DNA with specific enzymes at a constant temperature. After recognition, the released DNA or newly transcribed RNA initiates amplification; this technology can rapidly amplify TFBS-containing DNA strands, significantly improving detection effectiveness. In aTF biosensors, commonly used methods include rolled-circle amplification (RCA) [31] (Figure 2D), recombinase polymerase amplification (RPA) [31], strand displacement amplification (SDA) [25], and hybridization chain reaction (HCR) [25] (Figure 2C). For example, Cao et al. [31] combined the small-molecule recognition of an aTF with RCA and RPA, respectively, to detect the antibiotic TC and the food preservative 4-hydroxybenzoic acid (4-HBA) in food samples. Furthermore, in a study by Liu et al. [25], the recognition properties of TetR were combined with the signal amplification strategies of SDA and HCR. When TC is present, its binding to TetR causes its release from the TFBS, which initiates the SDA response. The released SDA product is activated by HCR to generate numerous G-quadruplexes. In the presence of ThT, these G-quadruplexes show intense fluorescence. This signal amplification strategy was able to detect TC in eight food samples with a detection limit of 17.61 ng/mL (Figure 3B).

#### 2.2.2. CRISPR-Cas Technology

The CRISPR-Cas system achieves signal amplification through its RNA-guided nuclease activity. This strategy converts the aTF-regulated transcriptional output into a specific CRISPR array RNA or DNA, which acts as a trigger to activate the non-specific collateral cleavage activity of Cas enzymes. Once activated, these enzymes indiscriminately cleave a large number of surrounding reporter probes, thereby transducing and amplifying a single recognition event into the cleavage of numerous reporter molecules [32] (Figure 2E). Among them, Cas12a and Cas13a are two different nuclease enzymes in the CRISPR-Cas system with some significant differences when used as signal amplification elements in biosensors. Cas12a mainly recognizes DNA targets; upon binding and cleaving a specific DNA target, it will activate its non-specific nuclease activity, which will cleave the surrounding ssDNA and achieve signal amplification. Cas13a, on the other hand, acts primarily on RNA targets, specifically binding to and cleaving the target RNA. For instance, Mahas et al. [26] assayed TC antibiotics based on the coupling of TetR-regulated in vitro transcription of CRISPR arrays with Cas12a activity. In the presence of TC, dissociation of *TetR* prompted in vitro transcription of CRISPR arrays, which in turn activated Cas12a to efficiently cleave fluorophore-quencher-labeled ssDNA, releasing fluorescent signals for signal amplification (Figure 3C). Lwasaki et al. [33] specifically triggered the RNase activity of Cas13a by in vitro-transcribed RNA sequences to cleave fluorophore-/quencher-labeled RNA, thus outputting fluorescence and achieving signal amplification.

#### 2.2.3. Nucleic Acid Modification

Unlike the previous two strategies, nucleic acid modification does not add an amplification step after the recognition event. Instead, it aims to enhance signal generation efficiency or the signal-to-noise ratio at the source by rationally engineering the components of the recognition module itself, including the TFBS or RNA aptamers. This involves site-directed mutagenesis, insertion, or deletion of DNA/RNA sequences to modulate their affinity for the aTF, stability, or conformation.

Base substitution allows for higher sensitivity and an appropriate linear detection range [34]. In contrast, point mutation is a feasible and flexible strategy that can be used to alter the LOD and linear detection range of aTF biosensors. Therefore, this strategy is preferred when optimizing aTF-based in vitro biosensors. Li et al. performed *hucO* and *otrO* mutations, based on mathematical modeling, to explore the effects of the number and position of point mutations on the aTF-TFBS affinity and ultimately obtained HucR and OtrR sensors with high sensitivity and a wide detection range [35].

The addition strategy is to add a few or several sequence bases to the TFBS sequence to optimize an aTF biosensor. For example, Sankar et al. [7] designed a DNA capable of binding SRTF1 to three TFBSs. The addition of three SRTF1 binding site strands increases the number of bound aTFs compared to one SRTF1 binding site strand. This is critical for electrochemical sensor design because an increase in the number of aTFs will result in a higher impedance change and thus increased sensitivity. The increase strategy can also be used to create new non-existing TFBSs using a computer design with human intervention. Dimas et al. [36] successfully developed a functional *ter-mphr* regulatory system by adding the operator spacer1 bp base to the middle of *tetO*. In addition, Schmidl et al. [37] demonstrated in their study that exchanging DBDs of regulators in the OmpR and NarL families leads to a reasonable change in the DNA-binding specificity of engineered proteins.

Deletion of non-essential nucleotides increases the binding affinity of aTFs, resulting in stronger and more reliable interactions and improved detection sensitivity and efficiency. Liu et al. [38] found that when truncated tetO was used in the construction of TC-based biosensors, the sensors exhibited lower basal activity and higher tetracycline induction rates and were therefore considered as materials for TC biosensors. Notably, this principle of enhancing performance through sequence truncation is equally applicable to aTF-RNA recognition systems. For instance, shortening the RNA aptamer can similarly improve the sensitivity of in vitro assays. Vockenhuber et al. [34], in their study on RNA aptamers containing DasR, employed the deletion of specific bases within the RNA sequences to enhance structural stability and effectively improve the signal-to-noise ratio. Furthermore, such sequence truncation also contributes to minimizing synthesis costs.

In short, in aTF-based biosensors for food contaminant monitoring, isothermal amplification technology currently represents the most widely adopted signal amplification strategy. Its key advantages include exponential amplification capability suitable for trace-level contaminant detection, as well as operation under constant temperature without the need for complex instrumentation, making it compatible with portable sensing platforms for on-site deployment. Although CRISPR-Cas technology offers exceptional target recognition specificity, it suffers from multi-step procedures and relatively high reagent costs compared to isothermal methods, limiting its suitability for large-scale monitoring applications. Moreover, nucleic acid modification serves as a crucial auxiliary enhancement strategy, in which optimization of TFBS or RNA aptamer structures through base substitution, sequence addition, or deletion strengthens the binding affinity between an aTF and its target, thereby improving signal output efficiency and detection reliability in subsequent amplification steps.

### 2.3. Signal Output System

The signal output system is responsible for transducing the amplified biochemical signals into physically detectable signals that are suitable for instrumental measurement or visual observation. These biochemical signals may include the abundance of specific nucleic acid sequences or the activation of enzyme cascades. The key to the system’s design lies in establishing an efficient and specific conversion pathway from these amplified products to final readouts such as light, electricity, and color. Currently, the signal output of most aTF-based in vitro biosensors is mainly in the form of fluorescence [39], colorimetric [40], bioluminescence [41] and electrical signals [42] (Figure 2F–I). Selecting the optimal modality requires a critical trade-off among key performance parameters to meet specific application needs. A study by Lopreside et al. [40] systematically compared fluorescence, colorimetric and bioluminescence reporters, underscoring the importance of this selection (Figure 3D). In general, fluorescent and bioluminescent systems achieve high sensitivity, their LODs are often in the range of pM to nM, and they are ideal for laboratory quantification, whereas colorimetric systems, with their rapid visual readouts within minutes yet moderate sensitivity in the micromolar range, are favored for on-site screening. Electrochemical methods, which provide responses within seconds to minutes, excel in portability, showing great promise for point-of-care devices. Notably, emerging mechanical signal transduction mechanisms like microcantilevers can reach parts-per-trillion levels but currently depend on specialized equipment, limiting field deployment. Ultimately, robustness against matrix interferences is a critical consideration for all systems. The following sections detail the principles and applications of each category, providing a basis for the consideration of these choices.

#### 2.3.1. Fluorescence

Fluorescent signal output systems convert aTF recognition into optical signals through different mechanisms, which can be broadly categorized into two classes, genetically encoded fluorescent reporters and exogenously introduced fluorescent probes, to meet varying demands for detection rapidity, sensitivity, and simplicity.

Genetically encoded fluorescent reporters rely on the transcription and expression of the sensing genetic circuit. Among these, fluorescent proteins, such as GFP and EmGFP, serve as conventional elements. Their detection mechanism is based on the target-induced expression of the protein, which spontaneously folds post-translation to form an intrinsic fluorescent chromophore, enabling quantification by measuring fluorescence intensity [39,43,44,45]. Redesigning their structures through protein engineering can further optimize their performance; for instance, the super-folded green fluorescent protein (sf GFP) [46], obtained by targeted mutagenesis, has more stable fluorescent properties, rapid folding capabilities, and resistance to interference from fusion proteins, making it an ideal reporter element [47].

Compared to fluorescent proteins that require the complete translation process, fluorescent RNAs offer a faster response pathway. Their core mechanism lies in the transcription of specific RNA aptamers that fold into precise three-dimensional structures, thereby specifically binding to and activating otherwise non-fluorescent dye molecules, leading to rapid fluorescent signal ‘turn-on’ [48] A representative application of this strategy is the work by Jung et al. [6], who utilized a three-way junction dimerized broccoli (3WJdB) RNA aptamer as a signal output element in combination with DFHBI-1T dye to successfully generate a visible and stable fluorescent system within minutes and used the system to create an aTF biosensor for detecting various water pollutants (Figure 3E).

Exogenously introduced fluorescent probes, in contrast, do not depend on the cellular gene expression machinery but generate or modulate fluorescence directly through physical or chemical interactions. Probe design based on fluorescence resonance energy transfer (FRET) is a key strategy in this category. Its working principle relies on distance-dependent non-radiative energy transfer between a donor, like a quantum dot (QD) [49], and an acceptor fluorophore. Chen et al. [50] cleverly combined a DNA-modified gold surface labeled with a thio-Cy5 fluorophore and TetR ligated with a QD. When anhydrous tetracycline (aTC) was added, the TetR-QD complex was released from the surface-bound DNA, leading to a significant attenuation of the FRET signal. This method’s detection time is only 32 min, and it is easy to perform and responsive. Furthermore, directly enhancing fluorescence through the specific binding of certain dyes to sensing reaction products constitutes another straightforward and efficient strategy. For example, ThT is a fluorescent dye that specifically binds to G-quadruplexes, thereby enhancing its own fluorescence [51]. This property can be exploited to respond to TC levels in food without labeling or modification, significantly reducing the complexity and cost of the assay [25].

#### 2.3.2. Colorimetry

The principle of colorimetry is to analyze a sample by detecting color changes caused by a specific substance which can be directly observed by the naked eye, offering advantages of operational simplicity and visual readout [52]. Its core lies in the use of biocatalysts to translate target recognition events into visible color signals, primarily through two types of catalytic systems which provide inherent signal amplification.

One system relies on the catalytic activity of natural enzymes. For example, β-galactosidase (LacZ) can be expressed as a reporter element. Upon activation, a single LacZ enzyme hydrolyzes numerous molecules of chromogenic substrates, such as chlorophenol red-β-D-galactopyranoside (CPRG), releasing a structurally altered chromophore (chlorophenol red) and thereby amplifying the initial recognition event into a pronounced color shift from yellow to purple [53]. Duyen et al. [28] utilized this enzymatic amplification mechanism to assess antibiotic concentrations by monitoring LacZ activity-mediated color change (Figure 3F).

The other system employs enzyme-mimicking nanomaterials, known as nanozymes, which often offer superior stability compared to their natural counterparts. A representative example is the G-quadruplex/hemin complex, which functions as a peroxidase nanozyme. It efficiently catalyzes the oxidation of colorless substrates (e.g., ABTS^2−^) by hydrogen peroxide (H_2_O_2_), generating a green oxidized product (ABTS·^−^). This continuous turnover of substrate molecules provides significant signal amplification, enabling stable and sensitive detection of various small molecules [54] and offering a clear visual threshold ideal for point-of-care applications.

Colorimetric biosensors are particularly suitable for preliminary screening in resource-limited settings, as they provide rapid visual readouts within minutes, without relying on complex or expensive lab equipment. However, their performance is constrained by limitations in signal intensity and visual resolution, typically restricting analysis sensitivity to the μM range with a narrow dynamic scope. Meanwhile, interference from colored or turbid sample matrices often restricts their application to qualitative or semi-quantitative analysis. Recently, studies have focused on optimizing the aTF-regulated signal generation process. Strategies such as coupling with nanozymes for signal amplification or integrating smartphone imaging for precise quantification aim to efficiently translate the specific recognition by aTFs into more sensitive and reliable visual signals, thereby extending their applicability in on-site quantitative detection.

#### 2.3.3. Bioluminescence

Bioluminescence is a biochemical reaction that generates light without an external excitation source, making it inherently resistant to autofluorescence interference from complex matrices and thus particularly suitable for analyzing colored samples like food. This output modality relies on luciferase enzymes, which catalyze light-emitting oxidation reactions with specific substrates. In aTF-based biosensors, the presence of the target analyte induces the expression of a luciferase reporter gene. The subsequent addition of the corresponding substrate yields a bioluminescence signal whose intensity provides a quantitative measure of the target concentration. The analytical performance of this system is highly dependent on the specific luciferase employed.

Among luciferases, firefly luciferase (LucFF) can be luminescent entirely by gene expression without the addition of exogenous luciferin. Pellinen et al. [41] used LucFF as a reporter to establish a cell-free biosensor based on MerR and TetR to detect Hg^2+^ and TC. Although it offers operational simplicity, the catalytic turnover rate of LucFF is generally moderate, leading to longer detection times and limited sensitivity in quantitative applications.

Subsequently, researchers found a luciferase called NanoLuc luciferase, which has higher activity and a smaller size than the conventional LucFF [55] and can quantify TC and erythromycin in milk samples more rapidly [29]. Compared to LucFF, NanoLuc exhibits significantly higher specific activity and greater physical stability, which can be directly translated into faster response kinetics, enabling it to complete quantitative analysis of antibiotics in milk within approximately 20 min, demonstrating a substantial improvement in speed. Moreover, the high signal intensity and robustness of NanoLuc make it highly compatible with miniaturized, portable detection formats. Its successful integration with smartphone-based readout systems highlights its strong potential for on-site, point-of-need testing in resource-limited environments. It is noteworthy that NanoLuc requires the addition of an exogenous synthetic substrate, like furimazine, whereas LucFF functions with its native luciferin.

Therefore, the choice between these two reporter systems involves careful consideration aligned with application priorities. LucFF provides a straightforward, self-contained system advantageous for proof-of-concept and standardized laboratory setups. In contrast, while NanoLuc introduces an additional step for substrate addition, it offers significant performance improvements in sensitivity, response speed, and adaptability to field-deployable formats, making it the preferred choice for next-generation biosensors requiring rapid, sensitive, and on-site detection capabilities.

#### 2.3.4. Electrical Signals

Electrochemical signal output is achieved by converting the biorecognition event into measurable changes in electrical parameters such as current, potential, or impedance. This output modality typically leverages portable electronic devices for signal transduction and readout. Nowadays, blood glucose meters, as sophisticated electronic devices, have been successfully applied for electrical signal output. Amalfitano et al. [42] established a method for TC detection mediated by blood glucose meters. In this approach, *TetR* regulates the expression of the reporter gene alginase in the presence of TC, which converts glucose-containing inert precursors into glucose monomers, thereby facilitating TC detection via measurement of glucose concentration using blood glucose meters (Figure 3G). Currently, there are relatively few studies on aTF biosensors with electrochemical reporter elements. Electrochemical analysis offers advantages such as miniaturization, rapid detection and low cost. Therefore, the development of novel aTF electrochemical sensors in the future will create significant opportunities for advancements in the field of food safety detection.

#### 2.3.5. Mechanical Signals

The conversion of biological signals into mechanical signals can promote the LOD, thus enabling accurate detection of ultra-trace substances. Cantilever beam-based reporters have been widely used for aTF and small-molecule interactions. Agarwal et al. [30] recently developed an aTF biosensor based on a microcantilever beam reporter. Modification of DNA containing the aTF operator on the surface of the microcantilever beam can cause the microcantilever beam to deflect. The presence of TC/Pb^2+^/Cd^2+^ molecules moves its corresponding aTF away from the TFBS, which leads to microcantilever de-bending. When the AFM detects the re-generated deflection, it can be converted to the target concentration. The entire reaction can be completed in 11 min, with tetracycline LOD reduced to 4 ppb, lead LOD reduced to 2 ppb, and cadmium LOD reduced to 1 ppb, which is more than two orders of magnitude lower than previous detections using WCBs or cell-free biosensors (Figure 3H).

In summary, among signal output modalities in aTF-based biosensors for food contaminant monitoring, fluorescence-based systems have achieved the highest degree of maturity and implementation. Whether delivered via proteins, RNA aptamers, or FRET, fluorescence readouts provide high sensitivity, low detection limits, and a wide dynamic range, allowing precise quantification of trace-level contaminants. These attributes establish fluorescence as the benchmark for laboratory-based analytical quantification. By contrast, electrochemical output systems offer compelling advantages for field deployment, including intrinsic miniaturization, rapid response, and direct compatibility with portable electronics. Their ability to function with minimal sample pretreatment positions them as a promising platform for on-site detection scenarios.

### 2.4. Sensing System

The design of the sensing system, as the cornerstone of the biosensor response, is crucial. In aTF-based in vitro biosensors, the sensing system mainly covers the compatibility buffer system and the cell-free system (Figure 2J–K). The compatibility buffer system provides a protective environment for biomolecules such as aTF, enzymes, etc., to prevent their inactivation and to ensure that the TFBS functions in the system. The cell-free system, on the other hand, mimics the living cell environment by synthesizing proteins in homogeneous buffer solution through in vitro DNA or mRNA templates, providing an ideal platform for biosensor research.

#### 2.4.1. aTF-TFBS Compatibility Buffer System

The aTF-TFBS compatibility buffer system is a solution composed of single components that can be supplemented with various substances based on the specific requirements of the assay. This design not only ensures compatibility with proteins and enzymes to avoid their inactivation during denaturation but also guarantees that the TFBS functions effectively within the system. On this basis, other components necessary for signal transduction can be incorporated to establish a comprehensive compatibility buffer system. When it is necessary to use the amplified luminescence proximity homogeneous assay (Alpha) technique, HBS-P buffer may be added to the system to minimize the background signal and maximize the signal-to-noise ratio [56]. Therefore, at present, for an aTF-TFBS-compatible buffer system, it is sufficient to simply add the required material components. However, due to its purer composition, the overall buffer system tends to have a higher cost.

#### 2.4.2. Cell-Free Systems

Cell-free systems are composed of DNA templates; cellular extracts, such as ribosomes, RNA polymerase, and protein factors; and necessary substrates like amino acids, peptides, energy sources, metabolic cofactors, etc. [19]. In the aTF cell-free biosensor, this includes a DNA template containing a TFBS, RNA polymerase, ribosomes, and the relevant aTF. Upon adding the target analyte to it, the corresponding aTF changes its affinity for the TFBS, which activates the expression of downstream reporter genes and completes the process of transcription or translation in vitro to achieve the signal output [57]. For example, the commonly used fluorescent protein reporter element can utilize ribosomes to ultimately translate the fluorescence of fluorescent proteins in a cell-free system. The RNA aptamer, on the other hand, only needs to be transcribed using RNA polymerase, aTF, and a DNA transcription template, and thus has the advantage of saving time. Selim et al. [58] constructed a biosensor for detecting putrescine in beef, used a cell-free system in which all the components existed, and utilized eGFP as the signal output. This system could detect 5.34 mM putrescine in 1 h. Despite the relatively complex composition of the cell extract in such a system, its overall construction cost is much lower compared to the aTF-TFBS-based compatible buffer system, so we currently prefer to use the cell-free system as the buffer system.

In summary, the rational engineering of high-performance aTF-based in vitro biosensors for on-site food monitoring hinges on the synergistic integration of four modular components. The recognition module achieves enhanced specificity for target contaminants and reduced matrix interference by employing directed evolution to modify the ligand-binding domains of aTFs, coupled with site-specific immobilization techniques. The signal amplification module significantly improves detection sensitivity by utilizing isothermal amplification technologies, supplemented with nucleic acid modifications, to achieve exponential amplification of target signals under complex conditions. The signal output module fulfills the requirements for on-site monitoring by prioritizing the integration of miniaturized, rapid-response, and portable detection devices to accomplish quantitative analysis of targets. The sensing system module ensures the repeatability and accuracy of the analysis process by establishing a stable reaction microenvironment through either a compatible buffer system or a cell-free system, supporting the efficient operation of all other modules.

The synergistic interplay of these four modules balances the analytical performance with practical utility, providing robust technical support for the real-time and on-site monitoring of food contaminants.

## 3. Application of aTF-Based In Vitro Biosensors in the Field of Food Safety

The successful development of aTF biosensors in vitro has given a new impetus to rapid and accurate analysis of food safety, attracting the attention of an increasing number of researchers. These sensors can effectively detect heavy metal ions, pesticide and veterinary drug residues, food additives, and foodborne pathogens in food. The aim of this chapter is to provide an overview of the current applications of aTF biosensors in vitro that have been successfully developed in the field of food safety to contribute to tackling the global food safety problem.

### 3.1. Detection of Heavy Metal Ions in Food by aTF-Based In Vitro Biosensors

Heavy metal pollution has become a widespread problem. Contaminated water containing heavy metal ions can lead to several health problems such as hepatotoxicity, renal failure and neurotoxicity [59]. According to the U.S. Environmental Protection Agency (EPA), the maximum contamination levels of Hg^2+^, Pb^2+^ and As^3+^ in drinking water are 10 nM, 72 nM and 130 nM, respectively [60,61,62]. Therefore, there is an urgent need to develop simple, sensitive and rapid methods to detect heavy metal ions in food. Currently, common aTFs for detecting heavy metal ions in food include MerR for Hg^2+^, PbrR for Pb^2+^, ArsR for As^3+^, etc.

**Figure 4:**
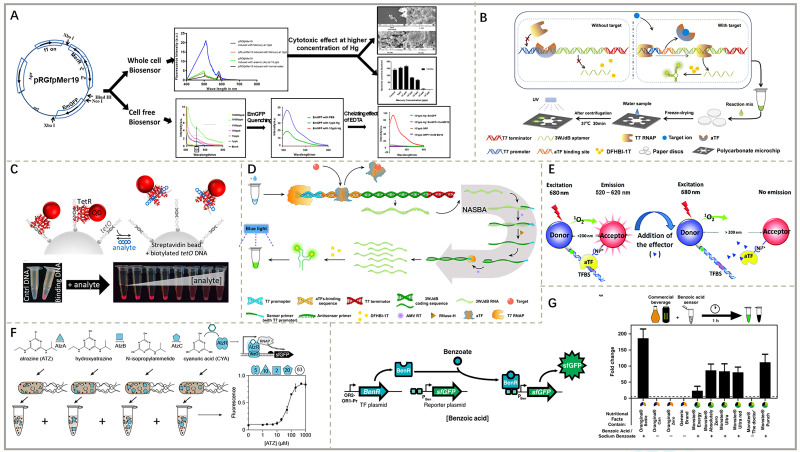
(**A**) Development of a MerR-based cell-free biosensor for the detection of Hg^2+^ using EmGFP as a reporter gene. Adapted from [39], with permission from the American Chemical Society, 2019. (**B**) Cell-free paper-based biosensor using aTF for the detection of heavy metal ions Hg^2+^ and Pb^2+^. Adapted from [63], with permission from Elsevier, 2022. (**C**) Modular fluorescent bead-based biosensor applied to detect TC in TetR-DNA-TC system. Adapted from [64], with permission from Wiley, 2020. (**D**) Cell-free biosensor based on NASBA signal amplification for the detection of tetracyclines and macrolides. Adapted from [65], with permission from *Analytical Methods*, 2009. (**E**) Fluorescent biosensor using OtrR and microbeads for the detection of hygromycin. Adapted from [35], with permission from the Royal Society of Chemistry, 2017. (**F**) Indirect detection of atrazine by modular-based cell-free biosensors. Adapted from [66], with permission from *ACS Synthetic Biology*, 2020. (**G**) Detection of benzoates in commercial beverages by a cell-free biosensor using BenR. Adapted from [67], with permission from *Nature Communications*, 2019.

MerR-based biosensors in vitro can specifically detect Hg^2+^ in food products. A study by Gupta et al. [39] showed that neutralizing pH and removing excess mercury using EDTA as a chelating agent significantly enhanced the fluorescence of EmGFP for the detection of Hg^2+^ using MerR fluorescent biosensors in a cell-free system. This approach resulted in a marked increase in assay sensitivity. Eventually, the LOD of this sensor could reach 1 ppb Hg^2+^ under the optimized conditions, meeting environmental standards for certain pollutants (Figure 4A). Gräwe et al. [47], on the other hand, prepared a paper-based fluorescent biosensor based on MerR through freeze-drying technology, which was combined with a smartphone camera and filters to detect the fluorescence intensity of sf GFP, effectively addressing on-site fluorescence measurement challenges while successfully detecting concentrations as low as 6 μg/L of Hg^2+^ in water.

PbrR-based in vitro biosensors can be used to detect Pb^2+^ in food. Zhang et al. [63] created an RNA fluorescent aptamer-based paper biosensor for simultaneous in situ detection of Hg^2+^ and Pb^2+^ in water using MerR and PbrR. In the presence of metal ions, the aTF detaches from the TFBS and initiates T7 RNAP to transcribe the 3WJdB aptamer. This aptamer binds to the non-fluorescent dye DFHBI-1T and folds into a three-dimensional structure to trigger fluorescence indicating the presence of target ions. After mounting the paper-based biosensor on a microchip, on-site detection could be completed within 1 h, with LODs of 0.5 nM for Hg^2+^ and 0.1 nM for Pb^2+^. In addition, the paper-based biosensor prepared by the team still worked properly after one month of freeze-drying, showing excellent stability at a cost of no more than ¥14.6 (Figure 4B).

ArsR-based in vitro biosensors can detect arsenic ions in food. Zhang et al. [42] constructed a biosensor that could detect mercury and arsenic, but the green fluorescent signal was observed after 3 h of reaction, and the LOD values for Hg^2+^ and Ar^3+^ were 661.005 nM and 1078.074 nM, respectively. If the sensor they designed is intended for use outside the laboratory environment, further development is still needed to obtain lower detection limits and faster response times. Wang et al. [68] designed an in vitro arsenic biosensor using a genetic circuit encoded by ArsR. After targeted evolutionary screening, the ArsR mutant ep3 was the most sensitive to arsenic, modulating the expression of GFP and showing about 3.4-fold fluorescence enhancement. Upon further optimization, its LOD could reach 3.65 μg/L. Lin et al. [53] prepared a paper-based biosensor using the colorimetric output as a sensing system, which could detect 0.5 μM arsenic ions within 3 h. In addition, the authors found that the use of sucrose as a lyophilization protectant could effectively address the problem of protein denaturation and loss of enzyme activity that occurs during drying and rehydration of paper.

### 3.2. Detection of Pesticide and Veterinary Drug Residues in Food by aTF-Based In Vitro Biosensors

Pesticides and veterinary drugs play a vital role in enhancing food production and animal husbandry. Nonetheless, their excessive or improper use results in residues in food and the environment. These residues pose significant threats to human health, ranging from acute poisoning to potential long-term risks such as cancer.

Therefore, accurate and timely detection of antibiotic residues in food is essential. At present, common aTFs used to identify veterinary drugs in food include TetR to identify tetracyclines, MphR to identify macrolides, and OtrR to identify hygromycin, and common aTFs used to identify pesticides in food include TtgR to identify carbaryl and AtzR to identify atrazine.

TetR-based in vitro biosensors can detect veterinary TC in food. In recent years, researchers have successively established a series of in vitro sensing platforms to detect TCs by combining isolated TetR with QD-based FRET readout technology [50,69]. For example, Chern et al. [64] used TetR-labeled QDs bound to beads modified with TetR homologous teto DNA sequences. Upon addition of the analyte anhydrous tetracycline (aTC), the TetR-QD complex was released from the beads, resulting in a significant decrease in the fluorescence of the beads, while the supernatant showed red fluorescence emitted by the QD. The sensor can be read by eye or digital camera within 5 min of analyte addition and has the advantage of being easy to assemble and simple to operate, without the need for a washing step or additional equipment (Figure 4C).

MphR-based in vitro biosensors can detect veterinary macrolides in food. In the study by Bi et al. [65], after activating transcription to produce 3WJdB RNA, the fluorescence signal could be enhanced 1.57–27-fold by using nucleic acid sequence-based amplification (NASBA). The LOD reached 0.01 μM for both aTC and erythromycin, achieving highly sensitive detection and rapid response to the target substances (Figure 4D). In addition, Rodriguez-Serrano et al. [70] reported a TMSD-mediated signal transduction and amplification mechanism to regulate the kinetics of the chain displacement reaction through the competition between an aTF and an endonuclease, and successfully constructed in vitro biosensors for TetR and MphR, which enabled the detection of tetracycline and macrolide at the nanomolar level in water in a few minutes. With the help of a smartphone, Zhang et al. [29] recorded the bioluminescence output of each sample using a smartphone camera, observed the dose-dependent bioluminescence response by simply browsing through the photographs, and quantified TC and erythromycin in milk within 15 min. The method visually identified safe and excessive milk samples.

OtrR-based in vitro biosensors can detect the veterinary drug hygromycin in food. In 2016, Li’s team, in ref. [35], constructed a biosensing platform for hygromycin for the first time by using OtrR as an in vitro recognition element and Alpha as a transduction element. In this platform, biotinylated DNA sequences containing a TFBS and his6-labeled OtrR were anchored in donor beads and acceptor beads, respectively. Under laser excitation, 1O^2^ produced by donor beads by excitation can diffuse to acceptor beads. The combination of OtrR and the TFBS shortens the distance between donor beads and acceptor beads, so that the energy can be transferred from 1O^2^ to acceptor beads, producing a broad luminescent signal. In contrast, when hygromycin is present, it prompts the dissociation of OtrR from the TFBS, resulting in the separation of donor beads from acceptor beads and the inability of 1O^2^ to diffuse to acceptor beads, leading to a weakened luminescence signal. Based on the change in luminescence signal, the final LOD for hygromycin can be as low as 0.03 nM (Figure 4E).

AtzR-based in vitro biosensors can detect the pesticide atrazine in food. Due to the limited ability of existing natural biosensors to directly detect contaminants such as pesticides, Silverman et al. [66] combined a cyanuric acid biosensor with the atrazine–cyanuric acid metabolic pathway. In a premixed bacterial extract containing metabolic enzymes and AtzR, atrazine was converted by the metabolic enzymes into cyanuric acid that could trigger transcriptional reactions, thereby activating downstream reporter genes and generating signals (Figure 4F). This approach effectively solved the problem of aTF limitation and extended the detection range of the cell-free biosensor. However, the sensor failed to meet the atrazine limit of 3 ppb set by the EPA.

TtgR-based in vitro biosensors can detect the pesticide carbaryl in food. Chen et al. [71] have developed an in vitro biosensor for the detection of the pesticide carbaryl, engineering TtgR to specifically recognize carbaryl. Upon binding to carbaryl, transcription is activated and the released operator DNA can be quantified by qPCR in vitro. The minimum detectable concentration of carbaryl is 1–10 nM.

### 3.3. Detection of Additives in Food by aTF-Based In Vitro Biosensors

Food additives can provide great sensory enjoyment and commercial convenience, not only helping to preserve food freshness, but also improving food quality. However, the dosage must be strictly controlled during use. Misuse of food additives may cause harm to human health [72]. Therefore, quantitative analysis of food additive levels is of great importance. Currently, common aTFs that respond to food additives include LldR, which responds to lactic acid; HosA, which responds to PHBA; and BenR, which responds to benzoic acid.

LldR-based in vitro biosensors can detect the additive lactic acid in food products. In the food industry, lactic acid is an important indicator of product freshness and stability, as well as the degree of fermentation, and it is also used as an acidulant, food preservative or flavoring agent in foods and beverages [73]. Lactic acid exists in two enantiomers, L-lactic acid and D-lactic acid. Xu et al. [74] constructed a fluorescent biosensor for L-lactic acid by using the STLldR in S. typhimurium LT2 as a recognition element for the specific sensing of L-lactic acid in conjunction with luminescence transduction-based FRET technology, with an LOD of 0.68 μM, which quantified the L-lactic acid in bacterial fermentation samples, enzymes and yoghurt. However, lactic acid is usually present as two stereoisomers in many biological samples. Therefore, a biosensor capable of detecting both D-lactic acid and L-lactic acid is needed. Xiao et al. [56] have developed for the first time an LldR-based biosensor capable of detecting the concentrations of D-lactic acid and L-lactic acid in fermentation samples at the same time, which concentrations are independent of the ratio of the two isomers. In addition, this biosensor was optimized by mutating the DNA sequence of the LldR binding site, which increased the LOD to 2.34 mM.

HosA-based in vitro biosensors can detect the preservative PHBA in food products. Liang et al. [75] developed an assay platform that combines aTF recognition with CRISPR-Cas12a. The platform converts small-molecule recognition signals into easily readable dsDNA signals via HosA. Free dsDNA binds to Cas12a-crRNA complexes and activates the non-specific ssDNA trans-cutting activity of Cas12a, which cuts the fluorescent bursting agent-labeled ssDNA probe. Based on the change in the fluorescence signal, TC can be analyzed qualitatively or quantitatively, with an LOD of 1.8 nM. Yao et al. [54] combined the small-molecule recognition of HosA with the SDA reaction and established both fluorescence and colorimetric methods for the detection of PHBA. The fluorescence method has an LOD of 1.35 nM, with a linear range of 5–300 nM, and the colorimetric method has an LOD of 2.55 nM, with a linear range of 10–300 nM.

BenR-based in vitro biosensors can detect the preservative benzoic acid in food. Benzoic acid is widely used in food and feed as an antimicrobial preservative [76]. Voyvodic et al. [69] developed a cell-free biosensor for the detection of benzoate. In the presence of benzoate, BenR binds to the PBen promoter and activates the expression of the sf GFP gene. The system was able to differentiate beverages containing benzoate with 100% accuracy (Figure 4G). Zhang et al. [42], on the other hand, developed an activated sensing system for the detection of benzoic acid which activates a transcriptional response through a complex of BenR and its cognate organic molecules, resulting in high expression of GFP. However, the sensitivity of this system for the detection of benzoic acid was low, and the authors suggested that this may be related to the peptide chain of the regulatory protein BenR, which is more difficult to synthesize in cell-free systems.

### 3.4. Detection of Pathogens in Food by aTF-Based In Vitro Biosensors

It is well known that foodborne pathogens, like bacteria, fungi, viruses, etc., are widely present in human daily life. They may contaminate food during food production, processing, storage and transport, rapidly triggering foodborne illnesses that result in a variety of symptoms such as headache, blurred vision and vomiting [77]. Therefore, early identification of foodborne pathogens is essential for preventing outbreaks and protecting individual health. Currently, LasR, a common identification component for detecting pathogens, specifically identifies *Pseudomonas aeruginosa* (Table 1).

Quorum sensing (QS) is a system by which bacteria communicate through chemical signals called self-inducers [78]. When the concentration of QS signaling molecules reaches a certain threshold, they are recognized by target receptors within the cell, which triggers transcriptional changes in certain genes, and this, in turn, triggers the simultaneous expression of related genes [79]. Gram-negative bacteria mainly utilize acyl homoserine lactones (AHLs) as QS molecules, while Gram-positive bacteria mainly use peptides. *P. aeruginosa* is a common food pathogenic microorganism. Wen et al. [80] designed a cell-free biosensor that utilizes the 3OC12-HSL (N-3-oxododecanoyl-L-homoserine lactone)-induced LasR system for the detection of QS molecules in respiratory samples of patients infected with *P. aeruginosa*, achieving an LOD of 4.9 nM. In addition, Seto et al. [81] developed a LasR-based agarose bead biosensor to monitor the growth of *Pseudomonas aeruginosa*. In the virulence mechanism, the production of 3OC12-HSL leads to the dissociation of LasR from the promoter, which activated the expression of virulence genes and the fluorescence signals of GFP reporter genes. Currently, aTF-based in vitro biosensors have not been applied in food matrices for pathogen detection. This translational gap arises from key technical hurdles imposed by the complex food environment. The primary barriers include: (1) receptor gaps, with existing aTF libraries being largely designed for targets like heavy metals or antibiotics, lacking specific elements to recognize the diverse metabolites produced by foodborne pathogens; (2) matrix interference, as food components such as proteins, fats, and endogenous enzymes can non-specifically adsorb or degrade sensor components, sequester target molecules, and introduce noise that destabilizes the signal; (3) standardization bottlenecks, the physicochemical diversity among different food matrices hindering the development of universal sample-preparation protocols, making validation costly and impractical for broad commercialization. Consequently, the translation of aTF-based biosensors into food safety testing remains in exploratory stages.

### 3.5. Detection of Other Contaminants in Food by aTF-Based In Vitro Biosensors

Biogenic amines are toxicological risks present in many foods. Among them, putrescine is the most common foodborne biogenic amine and is commonly used as a food quality control marker [82]. PuuR is an aTF capable of recognizing putrescine. The use of PuuR to construct WCBs can simplify food preprocessing and reduce costs. Selim et al. [58] re-optimized the puuO binding site in a recognition system and constructed an in vitro PuuR biosensor for the combined detection of putrescine with PuuR. The sensor was highly specific and sensitive (LOD = 5.37 mM) and could detect putrescine in beef samples that did not require temperature preservation. In addition, some illegally added substances can cause direct harm to human health and others can harm the rights of consumers. γ-Hydroxybutyric acid is a class of psychotropic substances, but it has been illegally mixed into beverages as a psychedelic substance [83]. High-performance liquid chromatography (HPLC), although capable of accurately detecting γ-hydroxybutyric acid, lacks timeliness and portability. Gräwe et al. [48] constructed a fluorescence-based in vitro BlcR biosensor using BlcR as a potential sensing element for γ-hydroxybutyric acid. Combined with a lyophilized paper base and a hand-held fluorescent shooter, the BlcR-based in vitro biosensor is capable of detecting γ-hydroxybutyric acid in beverages, providing direction for novel detection methods for food safety monitoring.

## 4. Summary and Outlook

To date, biosensors have become an important tool for rapid food safety detection. Notably, significant progress has been made in scientific research on the design and development of aTF-based in vitro biosensors, and these innovations are undoubtedly revolutionary and will play a key role in addressing global food safety challenges. Therefore, this paper presents a comprehensive review of the strategies for the construction of aTF-based in vitro biosensors; through precise engineering, clever signal amplification strategies and diverse output modes, biosensors can respond rapidly and accurately recognizing a variety of target molecules. However, given that the technology is still in the initial development stage, it faces many challenges. Further research and innovation are needed to advance this field.

Given that the technology remains in its early development stage, its translation from laboratory research to real-time applications and commercial adoption still faces several core technical limitations. First, to achieve commercialization, the laboratory selectivity of aTFs must be proven to be convertible into reliable specificity under complex real sample conditions, especially when facing the problems of cross-reactivity and matrix interference. Second, the limited stability of allosteric transcription factors hinders their performance under extreme temperature or pH conditions. Third, the incompatibility between response kinetics and signal amplification systems restricts rapid detection of trace-level targets. Fourth, high scaling-up costs, together with inadequate storage stability and operational convenience, constrain commercial implementation. These barriers currently impede broader deployment, underscoring the need for focused research and innovation to overcome these bottlenecks. Future studies should therefore prioritize the following directions:

First, improve specificity. Future research should focus on developing more selective recognition elements and optimizing sensor structures to better distinguish similar analytes. First, aTFs with high affinity and specificity for target molecules need to be selected as the core element of the sensor. With the advancement of gene editing technology, we can design highly specific aTFs that are specifically tailored to specific target molecules or pathogens. In addition, TFBSs are genetically engineered to be mutated to enhance their binding ability to specific targets while decreasing the binding ability to non-target molecules. The introduction of selective signaling pathways into the sensor is also key to ensuring that the signal is delivered and amplified only in the presence of the target molecule, thus effectively reducing background noise.

Second, improve stability. Some biosensors may need to operate in extreme environments, such as extremely high or low temperatures and extreme pH conditions. Therefore, improving the stability of aTFs can enable a sensor to remain effective in more diverse environments. The structure of aTFs can be optimized by structural biology and molecular dynamics simulations to enhance its stability. For example, stable disulfide bonds can be introduced or fusions can be effected using more stable homologous proteins. In addition, the aTF is encapsulated using polymer embedding, nanoparticles or other materials to protect the aTF from the external environment. At the same time, the sensor needs to be tested for long-term stability to determine its reliability in practical applications.

Third, commercialization and application promotion. With the continuous development in the fields of biomedicine and environmental monitoring, there is a growing demand for aTF biosensors for applications in disease diagnosis, drug development and environmental monitoring. Commercialization can meet this market demand and provide efficient and sensitive detection tools for related industries. Currently, paper-based biosensors constructed based on freeze-drying technology, whose activity can be preserved for a long period of time and can be activated by simply adding water when in use, have the advantages of simple operation and low cost, and thus are expected to achieve on-site detection. In addition, with the development of microfluidics and nanotechnology, miniaturized and portable biosensors can be developed, enabling real-time monitoring and rapid on-site analysis of environmental or human samples. At the same time, combined with artificial intelligence and big data analytics, data can be processed and analyzed in real time, thus improving the interpretation and prediction of results and promoting the commercialization and widespread application of biosensors.

## Figures and Tables

**Table 1:** Applications of aTF biosensors.

Application	Target	aTF	Signal Output	LOD	Detection Range	Detection Time	Application	Ref.
Heavy metal	Hg^2+^	MerR	Fluorescent	1 ppb	1–10^5^ ppb	1 h	Water	[39]
	Hg^2+^	MerR	Fluorescent	6 μg/L	——	1 h	Water	[47]
	Hg^2+^	MerR	Fluorescent	0.5 nM	0.5–500 nM	1 h	Experimental buffer system Testing: river, waste and seawater	[63]
	Pb^2+^	PbrR	Fluorescent	0.1 nM	1–250 nM	1 h	Experimental buffer system Testing: river, waste and seawater	[63]
	As^3+^	ArsR	Colorimetric	0.5 μM	1–100 μM	3 h	Water	[53]
	As^3+^	ArsR	Fluorescent	3.65 μg/L	0–50 μg/L	2.5 h	Water	[68]
Pesticide and veterinary drug residues	Tetracycline	TetR	RT-qPCR	1.73 nM	5–600 nM	~85 min	Milk	[31]
	Tetracycline	TetR	RPA	0.005 nM	0.01–1000 nM	~185 min	Milk	[31]
	Tetracycline	TetR	RCA	1.21 nM	10–300 nM	~75 min	Milk	[31]
	Tetracycline	TetR	Fluorescent	80 nM	0–200 µM	32 min	Analyte	[50]
	Tetracycline	TetR	Fluorescent	12.5 nM	12.5 nM–50 nM (visual)	5 min	Analyte Testing: Fetal Bovine Serum	[64]
	Tetracycline	TetR	Fluorescent	17.16 ng/mL	20–1000 ng/mL	2 h	Analyte Testing: water, milk, honey, chicken	[25]
	Tetracycline	TetR	Bioluminescence	10 ng/mL	30–500 ng/mL	90 min	Water	[41]
	Tetracycline	TetR	Bioluminescence	45 nM	0.75~2.5 μM	15 min	Milk	[29]
	Erythromycin	MphR	Bioluminescence	5.2 nM	30–300 nM	15 min	Milk	[29]
	Erythromycin	MphR	Fluorescent	100 nM	100–2500 nM	5–30 min	Pond water	[70]
	Erythromycin	MphR	Fluorescent	0.1 μM	0.1–15 μM	1 h	Milk	[65]
	Roxithromycin	MphR	Fluorescent	0.5 μM	0.5–15 μM	1 h	Milk	[65]
	Azithromycin	MphR	Fluorescent	0.5 μM	0.5–15 μM	1 h	Milk	[65]
	Clarithromycin	MphR	Fluorescent	0.1 μM	0.1–15 μM	1 h	Milk	[65]
	Oxytetracycline	OtrR	Fluorescent	0.03 nM	0.1–300 nM	~2 h	Analyte Testing: urine, serum	[35]
	Atrazine	AtzA	Fluorescent	20 μM	10–100 µM	1 h	Experimental buffer system Testing: tap and lake water	[66]
	Carbaryl	TtgR	qPCR	——	1–10 nM	~3 h	Experimental buffer system	[71]
Food additives	Lactic Acid	LldR	Fluorescent	0.68 μM	0.76–51.79 µM	20 min	Bacterial Fermentation Samples, Testing: fermented enzyme diluted samples and yogurt diluted samples	[74]
	Lactic Acid	LldR	Fluorescent	2.34 mM	1 μM–10 mM	2 h	Fermentation sample	[56]
	4-HBA	HosA	RT-qPCR	1.12 nM	5–300 nM	~85 min	River water	[31]
	4-HBA	HosA	RPA	0.0005 nM	0.001–10 nM	~185 min	River water	[31]
	4-HBA	HosA	RCA	1.73 nM	5–200 nM	~75 min	River water	[31]
	PHBA	HosA	Fluorescent	1.8 nM	9–180 nM	25 min	Serum	[75]
	PHBA	HosA	Fluorescent	1.35 nM	5–300 nM	——	Experimental buffer system	[54]
	PHBA	HosA	Colorimetric	2.55 nM	10–300 nM	——	Experimental buffer system	[54]
	Benzoic Acid	BenR	Fluorescent	1 μM	0–1 mM	1 h	Beverage	[67]
	Benzoic Acid	BenR	Fluorescent	21702.843 nmol/L	0–40 mM	16 h	Water	[42]

## Data Availability

The original contributions presented in this study are included in the article. Further inquiries can be directed to the corresponding authors.

## Abbreviations

The following abbreviations are used in this manuscript:

aTF	Allosteric transcription factor
ABTS	2,2′-azido-bis (3-ethylbenzothiazoline-6-sulfonic acid) diammonium salt
AHLs	Acyl homoserine lactones
DBD	DNA-binding domain
dsDNA	Double-stranded DNA
FRET	Fluorescence resonance energy transfer
GFP	Green fluorescent protein
EmGFP	Emerald green fluorescent protein
EPA	Environmental Protection Agency
HCR	Hybridization chain reaction
HPLC	High-performance liquid chromatography
LBD	Ligand-binding domain
LucFF	Firefly luciferase
NASBA	Nucleic acid sequence-based amplification
QDs	Quantum dots
QS	Quorum sensing
RCA	Rolled-circle amplification
RFP	Red fluorescent protein
RPA	Recombinase polymerase amplification
ssDNA	Single-stranded DNA
sf GFP	Super-folded green fluorescent protein
TC	Tetracycline
aTC	Anhydrous tetracycline
WCB	Whole-cell biosensor
YFP	Yellow fluorescent protein
3WJdB	Three-way junction dimerized broccoli
